# Crystal structure of the [(1,3-dimesityl-1*H*-imidazol-3-ium-2-yl)methano­lato]copper(II) chloride dimer: insertion of formaldehyde into a copper–carbene bond

**DOI:** 10.1107/S205698901801201X

**Published:** 2018-08-31

**Authors:** Christopher A. Dodds, Alan R. Kennedy

**Affiliations:** a WestCHEM, Department of Pure & Applied Chemistry, University of Strathclyde, 295 Cathedral Street, Glasgow G1 1XL, Scotland, UK

**Keywords:** crystal structure, copper, N-heterocyclic carbene, insertion, formaldehyde

## Abstract

The crystal structure of bis­[μ-(1,3-dimesityl-1*H*-imidazol-3-ium-2-yl)methano­lato-κ^2^ *O*:*O*]bis­[di­chlorido­copper(II)], [Cu_2_Cl_4_(C_22_H_26_N_2_O)_2_], is reported. The complex is assumed to have formed *via* the insertion of formaldehyde into the copper–carbon bond in an N-heterocyclic carbene complex of copper(I) chloride. The structure of the binuclear mol­ecule possesses a crystallographic­ally centrosymmetric Cu_2_O_2_ central core with the O atoms bridging between the Cu^II^ atoms and thus *Z*′ = 0.5. The copper centres are further ligated by two chloride ligands, resulting in the Cu^II^ atoms residing in a distorted square-planar environment. The Cu—O bond lengths are shorter than those previously reported in structures with the same central Cu_2_O_2_ motif. The complex displays C—H⋯Cl inter­actions involving the H atoms of the heterocycle backbone and the chloride ligands of a neighbouring mol­ecule.

## Abstract 2

The title complex is assumed to have formed *via* the insertion of formaldehyde into the copper–carbon bond in an N-heterocyclic carbene complex of copper(I) chloride·The Cu—O bond lengths are shorter than those previously reported in structures with the same central Cu_2_O_2_ motif. The complex displays C—H⋯Cl inter­actions involving the H atoms of the heterocycle backbone and the chloride ligands of a neighbouring mol­ecule.

## Chemical context   

The chemistry of N-heterocyclic carbene (NHC) ligands is prominent within the landscape of inorganic and organometallic chemistry and now, more than 25 years on from Arduengo’s land-mark paper (Arduengo *et al.*, 1991 ▸), this prominence looks set to continue. One area that has proven fruitful is the chemistry of NHCs with the group 11 transition metal elements. In particular, the chemistry with copper has resulted in an abundance of complexes that have proven to be effective catalysts for a range of organic transformations, including conjugate addition and carboxyl­ation (Egbert *et al.*, 2013 ▸). The preparation of neutral mono-NHC complexes with the general formulae [Cu(NHC)Cl] is routine and straightforward with a variety of synthetic routes to such species available (McLean *et al.*, 2010 ▸; Santoro *et al.*, 2013 ▸; Gibard *et al.*, 2013 ▸; Lake *et al.*, 2012 ▸). One of the simplest routes is the reaction of imidazol(in)ium chloride with Cu_2_O under reflux conditions with no requirement for the exclusion of air and water. The species formed are stable when they are isolated in the solid state but solutions show signs of oxidation upon standing for prolonged periods, especially when they are prepared in coordinating solvents such as THF or aceto­nitrile. The tell-tale green colour, which indicates the formation of copper(II) species, is surely a common observation for chemists who work with copper(I)–NHC species, but surprisingly the literature offers little on the identification of these species and corresponding reaction pathways. This is most likely a consequence of the inherent difficulty in characterizing the paramagnetic copper(II) species formed. Our inter­est in this area has previously revolved around the modification of [Cu(NHC)Cl] complexes through the replacement of the chloride ligand with the thio­cyanate ligand (Dodds & Kennedy, 2014 ▸). Herein we report the formation of the unusual (1,3-dimesityl-1*H*-imidazol-3-ium-2-yl) copper(II) chloride dimer, formed presumably from the insertion of formaldehyde into the Cu—NHC bond, upon prolonged standing of a THF solution containing [Cu(IMes)Cl] [IMes = 1,3-bis­(2,4,6-tri­methyl­phen­yl)imidazol-2-yl­idene] and trace amounts of formaldehyde at 255 K. The presence of formaldehyde was a result of the preparation of the imidazolium chloride precursor, which utilises paraformaldehyde. Evidently trace amounts of paraformaldehyde have been present during the reflux of Cu_2_O with imidazolium chloride, with the resulting solution generating the reported complex upon prolonged standing. This is the first structurally characterized example of a species formed through the insertion of a small mol­ecule into a copper—NHC bond. To date, all attempts to prepare the complex rationally have proven unsuccessful.
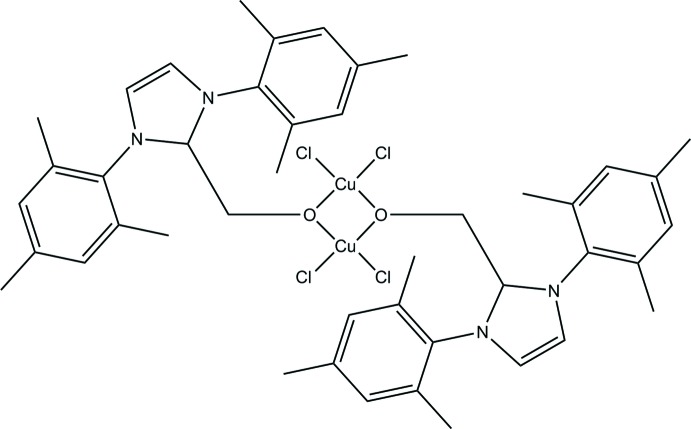


## Structural commentary   

**Figure 1:**
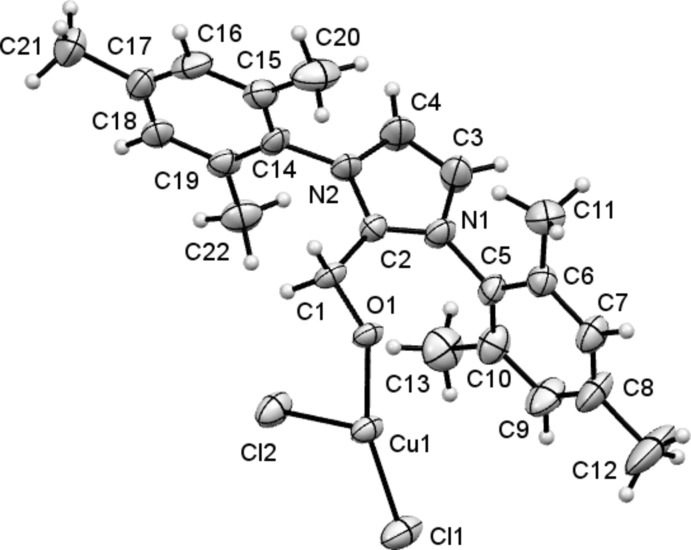
View of the contents of the asymmetric unit of (I). Non-H atoms are drawn as 50% probability ellipsoids and H atoms as small spheres of arbitrary size.

**Figure 2:**
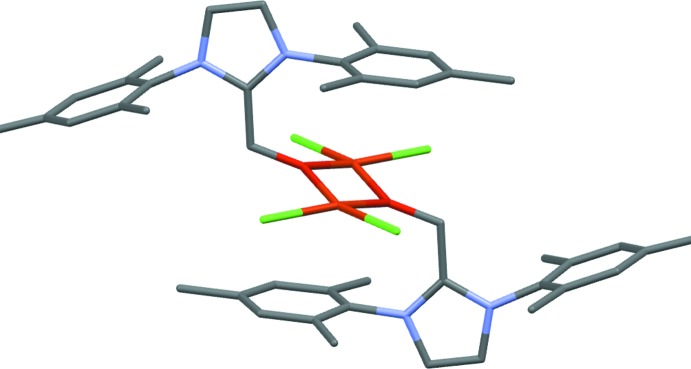
Mol­ecular structure of dimeric (I). The two halves of the dimer are related by −*x*, −*y* + 1, −*z* + 2. H atoms are omitted for clarity.

The structure of the binuclear mol­ecule (I) consists of a Cu_2_O_2_Cl_4_ central core, possessing a Cu_2_O_2_ four-membered ring with each copper centre further coordinated by two chloride ligands. Each dimer is sited around a crystallographic centre of symmetry and thus *Z*′ = 0.5. The structure of the asymmetric unit, with atom labels, is given in Fig. 1 ▸ and the dimeric unit is shown in Fig. 2 ▸. The copper centres reside in a distorted square-planar environment, as can be evidenced by the O—Cu—O and Cl—Cu—Cl bond angles [74.10 (11) and 97.58 (5)° respectively], which both deviate markedly from 90°. This distortion from ideal square planar geometry is further illustrated by the *trans* O—Cu—Cl bond angles [162.46 (10) and 162.36 (9)°], which also deviate noticeably from the expected 180 °. Similar Cu_2_O_2_Cl_4_ central cores have been observed previously by a number of groups (Schäfer *et al.*, 1965 ▸; Sager *et al.*, 1967 ▸; Watson & Johnson, 1971 ▸; Ivashevskaja *et al.*, 2002 ▸) with a similar distortion around the Cu^II^ atom observed. The Cu—O and Cu—Cl bond lengths are 1.934 (3) and 1.944 (3) Å and 2.2326 (13) and 2.2395 (12) Å, respectively. The Cu—O bond lengths are shorter than those observed in these previous reports [1.979–2.106 Å] while the Cu—Cl bond distances compare well to the previously reported examples [2.205–2.243 Å]. Finally, with regards to the central core, the Cu⋯Cu and O⋯O inter­nuclear distances are 3.0950 (12) and 2.337 (5) Å, respectively. The O⋯O distance is comparable to previous reports [2.366-2.591 Å] while the Cu⋯Cu distance is appreciably shorter when the comparison is made [3.190–3.245 Å]. The C—O bond length is 1.398 (4) Å, which is comparable with a structure previously reported by Hevia and co-workers (Uzelac *et al.*, 2016 ▸) in which the zwitterion [I*t*BuCH_2_OGa*R*_3_] [where I*t*Bu = 1,3-bis­(*tert*-but­yl)imidazol-2-yl­idene; *R* = tri­methyl­silylmeth­yl] displays a C—O bond length of 1.384 (3) Å. The new C—C bond formed has a bond length of 1.497 (6) Å, which again compares well with the equivalent bond in the aforementioned zwitterion [1.505 (3) Å]. The imidazolium ring is positioned such that it forms a dihedral angle of 90° with the plane of the Cu_2_O_2_ ring, torsion angle O1—C1—C2—N1 = 0.2 (7)°. This *syn* arrangement results in the C5–C10 mesityl rings lying above and below the Cu_2_O_2_ ring, as shown in Fig. 2 ▸. The distance between the centroids of the Cu_2_O_2_ ring and the mesityl ring is 3.390 (2) Å.

## Supra­molecular features   

**Figure 3:**
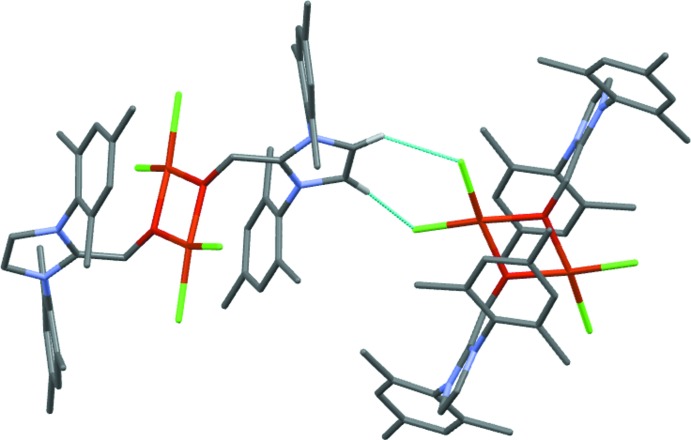
View highlighting the close Cl⋯H contacts between neighbouring mol­ecules of (I). See text for details.

The complex exhibits inter­molecular C—H⋯Cl inter­actions, specifically two short inter­actions between the H atoms on the unsaturated backbone of the heterocycle and the chloride ligands of a neighbouring mol­ecule at position −*x* − 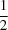, *y* + 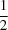, −*z* + 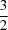. The inter­molecular H⋯Cl distances measure 2.51 and 2.76 Å. These inter­actions combine to give a two-dimensional supra­molecular motif than propagates parallel to the (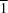
01) plane. Fig. 3 ▸ illustrates the C—H⋯Cl inter­molecular inter­actions and numerical details are given in Table 1 ▸.

## Database survey   

Outside the complex reported herein, there are eight structures reported in the Cambridge Structural Database (CSD, Version 5.39, update No. 2, February 2018; Groom *et al.*, 2016 ▸) that contain a Cu_2_O_2_Cl_4_ core and in which there is no additional coordination to the Cu^II^ atoms. The majority of structures reported contain pyridine *N*-oxide ligands (Schäfer *et al.*, 1965 ▸: refcodes CUCPYO, CUCPYO11 and CUCPYO13; Sager *et al.*, 1967 ▸: refcodes QQQBWD, QQQBWG and QQQBWJ; Watson & Johnson, 1971 ▸: refcode PHPYOC). The lone example that does not include a pyridine *N*-oxide ligand instead contains the related quinoline *N*-oxide ligand (Ivashevskaja *et al.*, 2002 ▸; refcode HULZOD). We are aware of no previous examples of ligands formed from NHC by an insertion reaction similar to the one reported herein.

## Synthesis and crystallization   

[Cu(IMes)Cl] was prepared according to literature procedures outlined by Abernethy and co-workers (McLean *et al.*, 2010 ▸). After isolation of an initial crop of [Cu(IMes)Cl], the filtrate was placed in the freezer (255 K) and left standing for ∼6 months. After this time the pale-orange THF solution had changed to a deep green and a small amount of green crystalline solid had precipitated alongside some green powder. This solid was isolated by filtration, yielding 34 mg of solid. The crystalline material isolated was suitable for single crystal X-ray diffraction. Additionally the isolated product was characterized by elemental analysis and ATR FT–IR.

Analysis calculated for C_44_H_52_N_4_O_2_Cl_4_Cu_2_: C, 56.38; H, 5.55; N, 5.98%. Found: C, 57.26; H, 5.64; N, 5.13%. ATR FT–IR: ν = 1502 (CO) cm^−1^.

## Refinement   

Crystal data, data collection and structure refinement details are summarized in Table 2 ▸. All H atoms were placed in calculated positions and refined in riding modes. C—H distances were 0.95, 0.99 and 0.98 Å for CH, CH_2_ and CH_3_ groups, respectively. For CH_3_ groups *U*_iso_(H) = 1.5*U*_eq_(C) and for all other types, *U*_iso_(H)_i_ = 1.2*U*_eq_(C).

## Supplementary Material

Crystal structure: contains datablock(s) I. DOI: 10.1107/S205698901801201X/pj2057sup1.cif

Structure factors: contains datablock(s) I. DOI: 10.1107/S205698901801201X/pj2057Isup2.hkl

CCDC reference: 1863799

Additional supporting information:  crystallographic information; 3D view; checkCIF report

## Figures and Tables

**Table 1:** Hydrogen-bond geometry (Å, °)

*D*—H⋯*A*	*D*—H	H⋯*A*	*D*⋯*A*	*D*—H⋯*A*
C1—H1*A*⋯Cl1^i^	0.99	2.59	3.216 (5)	121
C1—H1*B*⋯Cl2	0.99	2.54	3.215 (5)	126
C3—H3⋯Cl2^ii^	0.95	2.51	3.458 (6)	173
C4—H4⋯Cl1^ii^	0.95	2.76	3.385 (5)	124
C13—H13*A*⋯Cl2	0.98	2.79	3.684 (6)	152

Symmetry codes: (i) 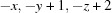; (ii) 
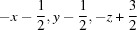
.

**Table 2:** Experimental details

Crystal data	
Chemical formula	[Cu_2_Cl_4_(C_22_H_26_N_2_O)_2_]
*M*_r_	937.77
Crystal system, space group	Monoclinic, *P*2_1_/*n*
Temperature (K)	123
*a*, *b*, *c* (Å)	11.1962 (8), 13.5321 (10), 15.5615 (8)
β (°)	107.705 (6)
*V* (Å^3^)	2246.0 (3)
*Z*	2
Radiation type	Mo *K*α
μ (mm^−1^)	1.23
Crystal size (mm)	0.10 × 0.08 × 0.05

Data collection	
Diffractometer	Oxford Diffraction Xcalibur E
Absorption correction	Multi-scan (*CrysAlis PRO*; Agilent, 2014 ▸)
*T*_min_, *T*_max_	0.993, 1.000
No. of measured, independent and observed [*I* > 2σ(*I*)] reflections	10241, 4960, 2814
*R*_int_	0.049
(sin θ/λ)_max_ (Å^−1^)	0.676

Refinement	
*R*[*F* ^2^ > 2σ(*F* ^2^)], *wR*(*F* ^2^), *S*	0.062, 0.144, 1.05
No. of reflections	4960
No. of parameters	259
H-atom treatment	H-atom parameters constrained
Δρ_max_, Δρ_min_ (e Å^−3^)	0.43, −0.32

Computer programs: *CrysAlis PRO* (Agilent, 2014 ▸), *SIR92* (Altomare *et al.*, 1994 ▸), *SHELXL2014* (Sheldrick, 2015 ▸) and *Mercury* (Macrae *et al.*, 2008 ▸).

## supplementary crystallographic information

### Crystal data

**Table:**

[Cu_2_Cl_4_(C_22_H_26_N_2_O)_2_]	*F*(000) = 972
*M**_r_* = 937.77	*D*_x_ = 1.387 Mg m^−^^3^
Monoclinic, *P*2_1_/*n*	Mo *K*α radiation, λ = 0.71073 Å
*a* = 11.1962 (8) Å	Cell parameters from 2164 reflections
*b* = 13.5321 (10) Å	θ = 3.0–28.7°
*c* = 15.5615 (8) Å	µ = 1.23 mm^−^^1^
β = 107.705 (6)°	*T* = 123 K
*V* = 2246.0 (3) Å^3^	Prism, green
*Z* = 2	0.10 × 0.08 × 0.05 mm

### Data collection

**Table:**

Oxford Diffraction Xcalibur E diffractometer	2814 reflections with *I* > 2σ(*I*)
Radiation source: sealed tube	*R*_int_ = 0.049
ω scans	θ_max_ = 28.7°, θ_min_ = 3.0°
Absorption correction: multi-scan (CrysAlis PRO; Agilent, 2014)	*h* = −14→14
*T*_min_ = 0.993, *T*_max_ = 1.000	*k* = −18→16
10241 measured reflections	*l* = −19→19
4960 independent reflections	

### Refinement

**Table:**

Refinement on *F*^2^	0 restraints
Least-squares matrix: full	Hydrogen site location: inferred from neighbouring sites
*R*[*F*^2^ > 2σ(*F*^2^)] = 0.062	H-atom parameters constrained
*wR*(*F*^2^) = 0.144	*w* = 1/[σ^2^(*F*_o_^2^) + (0.0526*P*)^2^ + 0.3159*P*] where *P* = (*F*_o_^2^ + 2*F*_c_^2^)/3
*S* = 1.05	(Δ/σ)_max_ = 0.001
4960 reflections	Δρ_max_ = 0.43 e Å^−^^3^
259 parameters	Δρ_min_ = −0.32 e Å^−^^3^

### Special details

**Table:**

Geometry. All esds (except the esd in the dihedral angle between two l.s. planes) are estimated using the full covariance matrix. The cell esds are taken into account individually in the estimation of esds in distances, angles and torsion angles; correlations between esds in cell parameters are only used when they are defined by crystal symmetry. An approximate (isotropic) treatment of cell esds is used for estimating esds involving l.s. planes.

### Fractional atomic coordinates and isotropic or equivalent isotropic displacement parameters (Å^2^)

**Table:**

	*x*	*y*	*z*	*U*_iso_*/*U*_eq_	
Cu1	−0.06291 (5)	0.59936 (4)	0.96249 (3)	0.03601 (19)	
Cl1	−0.20608 (13)	0.68984 (10)	1.00019 (8)	0.0548 (4)	
Cl2	−0.03916 (12)	0.69819 (10)	0.85340 (7)	0.0489 (4)	
O1	0.0280 (3)	0.4888 (2)	0.93419 (16)	0.0318 (7)	
N1	−0.1319 (3)	0.3733 (3)	0.7821 (2)	0.0399 (10)	
N2	−0.0002 (4)	0.3953 (3)	0.7078 (2)	0.0398 (10)	
C1	0.0662 (4)	0.4767 (4)	0.8572 (2)	0.0333 (11)	
H1A	0.1493	0.4442	0.8747	0.040*	
H1B	0.0754	0.5426	0.8324	0.040*	
C2	−0.0235 (4)	0.4165 (3)	0.7853 (3)	0.0321 (11)	
C3	−0.1780 (5)	0.3228 (4)	0.7013 (3)	0.0551 (15)	
H3	−0.2531	0.2852	0.6824	0.066*	
C4	−0.0958 (5)	0.3371 (4)	0.6549 (3)	0.0585 (16)	
H4	−0.1024	0.3119	0.5967	0.070*	
C5	−0.1998 (4)	0.3750 (4)	0.8489 (3)	0.0350 (11)	
C6	−0.1849 (4)	0.2964 (4)	0.9081 (3)	0.0396 (12)	
C7	−0.2554 (5)	0.2987 (4)	0.9679 (3)	0.0476 (13)	
H7	−0.2456	0.2469	1.0108	0.057*	
C8	−0.3382 (5)	0.3731 (5)	0.9669 (3)	0.0604 (16)	
C9	−0.3530 (5)	0.4476 (4)	0.9046 (3)	0.0582 (15)	
H9	−0.4104	0.4996	0.9038	0.070*	
C10	−0.2855 (5)	0.4490 (4)	0.8423 (3)	0.0470 (13)	
C11	−0.1024 (5)	0.2100 (4)	0.9088 (3)	0.0574 (15)	
H11A	−0.0598	0.1905	0.9712	0.086*	
H11B	−0.0399	0.2280	0.8790	0.086*	
H11C	−0.1532	0.1548	0.8765	0.086*	
C12	−0.4118 (6)	0.3732 (6)	1.0350 (4)	0.104 (3)	
H12A	−0.3942	0.3123	1.0706	0.156*	
H12B	−0.5018	0.3771	1.0028	0.156*	
H12C	−0.3868	0.4303	1.0751	0.156*	
C13	−0.3075 (5)	0.5289 (4)	0.7712 (3)	0.0633 (16)	
H13A	−0.2279	0.5618	0.7755	0.095*	
H13B	−0.3672	0.5773	0.7808	0.095*	
H13C	−0.3415	0.4992	0.7113	0.095*	
C14	0.1027 (4)	0.4299 (4)	0.6795 (3)	0.0385 (12)	
C15	0.2083 (5)	0.3714 (4)	0.6951 (3)	0.0460 (13)	
C16	0.3012 (5)	0.4043 (4)	0.6583 (3)	0.0532 (14)	
H16	0.3751	0.3659	0.6670	0.064*	
C17	0.2875 (5)	0.4917 (4)	0.6093 (3)	0.0492 (14)	
C18	0.1831 (5)	0.5488 (4)	0.5989 (3)	0.0433 (13)	
H18	0.1762	0.6097	0.5674	0.052*	
C19	0.0875 (4)	0.5205 (4)	0.6326 (3)	0.0376 (12)	
C20	0.2251 (6)	0.2775 (4)	0.7500 (4)	0.0688 (18)	
H20A	0.2424	0.2941	0.8140	0.103*	
H20B	0.2955	0.2396	0.7419	0.103*	
H20C	0.1484	0.2378	0.7299	0.103*	
C21	0.3850 (5)	0.5198 (5)	0.5650 (4)	0.076 (2)	
H21A	0.3568	0.4996	0.5015	0.115*	
H21B	0.4643	0.4867	0.5959	0.115*	
H21C	0.3975	0.5916	0.5687	0.115*	
C22	−0.0275 (5)	0.5833 (4)	0.6182 (3)	0.0524 (15)	
H22A	−0.0312	0.6085	0.6764	0.079*	
H22B	−0.1023	0.5435	0.5900	0.079*	
H22C	−0.0240	0.6390	0.5787	0.079*	

### Atomic displacement parameters (Å^2^)

**Table:**

	*U*^11^	*U*^22^	*U*^33^	*U*^12^	*U*^13^	*U*^23^
Cu1	0.0437 (4)	0.0389 (4)	0.0313 (3)	0.0073 (3)	0.0201 (2)	0.0036 (3)
Cl1	0.0691 (9)	0.0562 (9)	0.0510 (7)	0.0252 (8)	0.0361 (7)	0.0140 (7)
Cl2	0.0570 (8)	0.0511 (8)	0.0478 (7)	0.0139 (7)	0.0297 (6)	0.0161 (6)
O1	0.0394 (18)	0.0363 (18)	0.0253 (14)	0.0041 (15)	0.0180 (13)	−0.0012 (14)
N1	0.042 (2)	0.053 (3)	0.0291 (19)	−0.008 (2)	0.0179 (17)	−0.0087 (19)
N2	0.048 (2)	0.047 (3)	0.0303 (19)	−0.012 (2)	0.0217 (17)	−0.0134 (19)
C1	0.037 (3)	0.041 (3)	0.026 (2)	0.004 (2)	0.0169 (19)	−0.001 (2)
C2	0.040 (3)	0.032 (3)	0.029 (2)	−0.002 (2)	0.0169 (19)	−0.001 (2)
C3	0.060 (4)	0.070 (4)	0.039 (3)	−0.030 (3)	0.021 (2)	−0.018 (3)
C4	0.073 (4)	0.074 (4)	0.037 (3)	−0.029 (3)	0.029 (3)	−0.023 (3)
C5	0.027 (2)	0.044 (3)	0.036 (2)	−0.004 (2)	0.0137 (19)	−0.001 (2)
C6	0.036 (3)	0.045 (3)	0.040 (3)	−0.007 (2)	0.015 (2)	−0.005 (2)
C7	0.050 (3)	0.054 (4)	0.046 (3)	0.001 (3)	0.025 (2)	0.012 (3)
C8	0.054 (4)	0.081 (5)	0.062 (3)	0.009 (3)	0.041 (3)	0.013 (3)
C9	0.050 (3)	0.064 (4)	0.072 (4)	0.023 (3)	0.035 (3)	0.016 (3)
C10	0.043 (3)	0.059 (4)	0.040 (3)	0.001 (3)	0.014 (2)	0.011 (3)
C11	0.061 (4)	0.054 (4)	0.064 (3)	0.001 (3)	0.028 (3)	−0.009 (3)
C12	0.100 (5)	0.136 (7)	0.114 (5)	0.040 (5)	0.089 (5)	0.037 (5)
C13	0.070 (4)	0.062 (4)	0.057 (3)	0.010 (3)	0.018 (3)	0.017 (3)
C14	0.041 (3)	0.053 (3)	0.028 (2)	−0.012 (3)	0.021 (2)	−0.012 (2)
C15	0.061 (4)	0.041 (3)	0.042 (3)	0.000 (3)	0.026 (2)	−0.006 (2)
C16	0.051 (3)	0.063 (4)	0.054 (3)	0.011 (3)	0.027 (3)	−0.014 (3)
C17	0.052 (3)	0.061 (4)	0.043 (3)	−0.021 (3)	0.027 (2)	−0.015 (3)
C18	0.058 (3)	0.046 (3)	0.032 (2)	−0.011 (3)	0.023 (2)	−0.010 (2)
C19	0.045 (3)	0.047 (3)	0.024 (2)	−0.002 (3)	0.014 (2)	−0.008 (2)
C20	0.096 (5)	0.054 (4)	0.064 (3)	0.028 (4)	0.036 (3)	0.004 (3)
C21	0.072 (4)	0.082 (5)	0.097 (4)	−0.031 (4)	0.058 (4)	−0.029 (4)
C22	0.061 (4)	0.062 (4)	0.034 (3)	0.003 (3)	0.016 (2)	−0.006 (3)

### Geometric parameters (Å, º)

**Table:**

Cu1—O1	1.934 (3)	C11—H11A	0.9800
Cu1—O1^i^	1.944 (3)	C11—H11B	0.9800
Cu1—Cl1	2.2326 (13)	C11—H11C	0.9800
Cu1—Cl2	2.2395 (12)	C12—H12A	0.9800
O1—C1	1.398 (4)	C12—H12B	0.9800
O1—Cu1^i^	1.944 (3)	C12—H12C	0.9800
N1—C2	1.334 (5)	C13—H13A	0.9800
N1—C3	1.386 (6)	C13—H13B	0.9800
N1—C5	1.463 (5)	C13—H13C	0.9800
N2—C2	1.341 (5)	C14—C15	1.381 (7)
N2—C4	1.381 (6)	C14—C19	1.410 (7)
N2—C14	1.432 (5)	C15—C16	1.404 (7)
C1—C2	1.497 (6)	C15—C20	1.511 (7)
C1—H1A	0.9900	C16—C17	1.390 (7)
C1—H1B	0.9900	C16—H16	0.9500
C3—C4	1.345 (6)	C17—C18	1.369 (7)
C3—H3	0.9500	C17—C21	1.507 (6)
C4—H4	0.9500	C18—C19	1.383 (6)
C5—C10	1.368 (6)	C18—H18	0.9500
C5—C6	1.383 (6)	C19—C22	1.502 (6)
C6—C7	1.392 (6)	C20—H20A	0.9800
C6—C11	1.488 (7)	C20—H20B	0.9800
C7—C8	1.365 (7)	C20—H20C	0.9800
C7—H7	0.9500	C21—H21A	0.9800
C8—C9	1.374 (7)	C21—H21B	0.9800
C8—C12	1.528 (6)	C21—H21C	0.9800
C9—C10	1.398 (6)	C22—H22A	0.9800
C9—H9	0.9500	C22—H22B	0.9800
C10—C13	1.513 (7)	C22—H22C	0.9800
			
O1—Cu1—O1^i^	74.10 (11)	C6—C11—H11C	109.5
O1—Cu1—Cl1	162.46 (10)	H11A—C11—H11C	109.5
O1^i^—Cu1—Cl1	95.69 (9)	H11B—C11—H11C	109.5
O1—Cu1—Cl2	95.60 (8)	C8—C12—H12A	109.5
O1^i^—Cu1—Cl2	162.36 (9)	C8—C12—H12B	109.5
Cl1—Cu1—Cl2	97.58 (5)	H12A—C12—H12B	109.5
C1—O1—Cu1	127.2 (3)	C8—C12—H12C	109.5
C1—O1—Cu1^i^	126.8 (3)	H12A—C12—H12C	109.5
Cu1—O1—Cu1^i^	105.90 (11)	H12B—C12—H12C	109.5
C2—N1—C3	109.5 (4)	C10—C13—H13A	109.5
C2—N1—C5	129.0 (4)	C10—C13—H13B	109.5
C3—N1—C5	121.5 (4)	H13A—C13—H13B	109.5
C2—N2—C4	109.3 (4)	C10—C13—H13C	109.5
C2—N2—C14	127.0 (4)	H13A—C13—H13C	109.5
C4—N2—C14	123.6 (3)	H13B—C13—H13C	109.5
O1—C1—C2	113.2 (3)	C15—C14—C19	123.5 (4)
O1—C1—H1A	108.9	C15—C14—N2	119.0 (5)
C2—C1—H1A	108.9	C19—C14—N2	117.5 (4)
O1—C1—H1B	108.9	C14—C15—C16	116.6 (5)
C2—C1—H1B	108.9	C14—C15—C20	122.3 (5)
H1A—C1—H1B	107.8	C16—C15—C20	121.1 (5)
N1—C2—N2	107.2 (4)	C17—C16—C15	121.4 (5)
N1—C2—C1	131.5 (3)	C17—C16—H16	119.3
N2—C2—C1	121.3 (4)	C15—C16—H16	119.3
C4—C3—N1	106.8 (4)	C18—C17—C16	119.6 (5)
C4—C3—H3	126.6	C18—C17—C21	120.9 (5)
N1—C3—H3	126.6	C16—C17—C21	119.4 (5)
C3—C4—N2	107.2 (4)	C17—C18—C19	122.0 (5)
C3—C4—H4	126.4	C17—C18—H18	119.0
N2—C4—H4	126.4	C19—C18—H18	119.0
C10—C5—C6	123.5 (4)	C18—C19—C14	116.9 (5)
C10—C5—N1	117.6 (4)	C18—C19—C22	120.9 (5)
C6—C5—N1	118.5 (4)	C14—C19—C22	122.2 (4)
C5—C6—C7	116.8 (5)	C15—C20—H20A	109.5
C5—C6—C11	123.9 (4)	C15—C20—H20B	109.5
C7—C6—C11	119.3 (5)	H20A—C20—H20B	109.5
C8—C7—C6	121.9 (5)	C15—C20—H20C	109.5
C8—C7—H7	119.0	H20A—C20—H20C	109.5
C6—C7—H7	119.0	H20B—C20—H20C	109.5
C7—C8—C9	119.1 (4)	C17—C21—H21A	109.5
C7—C8—C12	120.0 (5)	C17—C21—H21B	109.5
C9—C8—C12	120.9 (6)	H21A—C21—H21B	109.5
C8—C9—C10	121.6 (5)	C17—C21—H21C	109.5
C8—C9—H9	119.2	H21A—C21—H21C	109.5
C10—C9—H9	119.2	H21B—C21—H21C	109.5
C5—C10—C9	117.0 (5)	C19—C22—H22A	109.5
C5—C10—C13	122.1 (4)	C19—C22—H22B	109.5
C9—C10—C13	120.9 (5)	H22A—C22—H22B	109.5
C6—C11—H11A	109.5	C19—C22—H22C	109.5
C6—C11—H11B	109.5	H22A—C22—H22C	109.5
H11A—C11—H11B	109.5	H22B—C22—H22C	109.5
			
Cu1—O1—C1—C2	−95.8 (4)	C7—C8—C9—C10	0.1 (9)
Cu1^i^—O1—C1—C2	87.6 (4)	C12—C8—C9—C10	179.5 (6)
C3—N1—C2—N2	0.6 (5)	C6—C5—C10—C9	5.5 (7)
C5—N1—C2—N2	−178.9 (4)	N1—C5—C10—C9	177.7 (5)
C3—N1—C2—C1	−178.0 (5)	C6—C5—C10—C13	−174.4 (5)
C5—N1—C2—C1	2.5 (8)	N1—C5—C10—C13	−2.1 (7)
C4—N2—C2—N1	−0.3 (6)	C8—C9—C10—C5	−2.9 (8)
C14—N2—C2—N1	176.7 (5)	C8—C9—C10—C13	177.0 (5)
C4—N2—C2—C1	178.5 (4)	C2—N2—C14—C15	95.2 (6)
C14—N2—C2—C1	−4.5 (7)	C4—N2—C14—C15	−88.2 (6)
O1—C1—C2—N1	0.2 (7)	C2—N2—C14—C19	−88.1 (5)
O1—C1—C2—N2	−178.3 (4)	C4—N2—C14—C19	88.6 (6)
C2—N1—C3—C4	−0.7 (6)	C19—C14—C15—C16	−2.6 (7)
C5—N1—C3—C4	178.9 (5)	N2—C14—C15—C16	173.9 (4)
N1—C3—C4—N2	0.5 (7)	C19—C14—C15—C20	176.5 (4)
C2—N2—C4—C3	−0.1 (7)	N2—C14—C15—C20	−7.0 (7)
C14—N2—C4—C3	−177.3 (5)	C14—C15—C16—C17	0.5 (7)
C2—N1—C5—C10	90.0 (6)	C20—C15—C16—C17	−178.6 (5)
C3—N1—C5—C10	−89.4 (6)	C15—C16—C17—C18	2.0 (7)
C2—N1—C5—C6	−97.3 (6)	C15—C16—C17—C21	−175.3 (4)
C3—N1—C5—C6	83.2 (6)	C16—C17—C18—C19	−2.6 (7)
C10—C5—C6—C7	−5.0 (7)	C21—C17—C18—C19	174.7 (4)
N1—C5—C6—C7	−177.2 (4)	C17—C18—C19—C14	0.6 (6)
C10—C5—C6—C11	172.9 (5)	C17—C18—C19—C22	−178.4 (4)
N1—C5—C6—C11	0.7 (7)	C15—C14—C19—C18	2.1 (6)
C5—C6—C7—C8	1.9 (8)	N2—C14—C19—C18	−174.5 (4)
C11—C6—C7—C8	−176.1 (5)	C15—C14—C19—C22	−178.9 (4)
C6—C7—C8—C9	0.4 (9)	N2—C14—C19—C22	4.5 (6)
C6—C7—C8—C12	−179.0 (6)		

Symmetry code: (i) −*x*, −*y*+1, −*z*+2.

### Hydrogen-bond geometry (Å, º)

**Table:**

*D*—H···*A*	*D*—H	H···*A*	*D*···*A*	*D*—H···*A*
C1—H1*A*···Cl1^i^	0.99	2.59	3.216 (5)	121
C1—H1*B*···Cl2	0.99	2.54	3.215 (5)	126
C3—H3···Cl2^ii^	0.95	2.51	3.458 (6)	173
C4—H4···Cl1^ii^	0.95	2.76	3.385 (5)	124
C13—H13*A*···Cl2	0.98	2.79	3.684 (6)	152

Symmetry codes: (i) −*x*, −*y*+1, −*z*+2; (ii) −*x*−1/2, *y*−1/2, −*z*+3/2.
